# Improving Soil Resource Uptake by Plants Through Capitalizing on Synergies Between Root Architecture and Anatomy and Root-Associated Microorganisms

**DOI:** 10.3389/fpls.2022.827369

**Published:** 2022-03-09

**Authors:** Tania Galindo-Castañeda, Jonathan P. Lynch, Johan Six, Martin Hartmann

**Affiliations:** ^1^Sustainable Agroecosystems, Institute of Agricultural Sciences, Department of Environmental System Science, ETH Zürich, Zurich, Switzerland; ^2^Department of Plant Science, The Pennsylvania State University, University Park, PA, United States

**Keywords:** root anatomy and architecture, soil resource acquisition, endosphere and rhizosphere, microbial habitat, agriculture

## Abstract

Root architectural and anatomical phenotypes are highly diverse. Specific root phenotypes can be associated with better plant growth under low nutrient and water availability. Therefore, root ideotypes have been proposed as breeding targets for more stress-resilient and resource-efficient crops. For example, root phenotypes that correspond to the *Topsoil Foraging* ideotype are associated with better plant growth under suboptimal phosphorus availability, and root phenotypes that correspond to the *Steep, Cheap and Deep* ideotype are linked to better performance under suboptimal availability of nitrogen and water. We propose that natural variation in root phenotypes translates into a diversity of different niches for microbial associations in the rhizosphere, rhizoplane and root cortex, and that microbial traits could have synergistic effects with the beneficial effect of specific root phenotypes. Oxygen and water content, carbon rhizodeposition, nutrient availability, and root surface area are all factors that are modified by root anatomy and architecture and determine the structure and function of the associated microbial communities. Recent research results indicate that root characteristics that may modify microbial communities associated with maize include aerenchyma, rooting angle, root hairs, and lateral root branching density. Therefore, the selection of root phenotypes linked to better plant growth under specific edaphic conditions should be accompanied by investigating and selecting microbial partners better adapted to each set of conditions created by the corresponding root phenotype. Microbial traits such as nitrogen transformation, phosphorus solubilization, and water retention could have synergistic effects when correctly matched with promising plant root ideotypes for improved nutrient and water capture. We propose that elucidation of the interactive effects of root phenotypes and microbial functions on plant nutrient and water uptake offers new opportunities to increase crop yields and agroecosystem sustainability.

## Introduction

Global agriculture would greatly benefit from improvements in crop adaptation to limited water and nutrient availability. Novel strategies are urgently needed to develop crops that can acquire water and nutrients at higher than current rates (Ladha et al., 2005; Syers et al., 2008). Selecting cultivars with specific root architecture (Lynch, 2021) and anatomy (Lynch et al., 2021) as well as engineering microbiomes associated with crop species (Compant et al., 2019) are possible avenues to help develop technological innovations to improve soil resource acquisition. Microbes inhabiting the root endosphere and rhizosphere are important for crop production because they are primary drivers of C and nutrient cycling, as well as plant protection to biotic and abiotic stress (Compant et al., 2019). Moreover, root microbes may facilitate plant nutrient uptake (Jacoby et al., 2017), compete with plants for nutrients (Kuzyakov and Xu, 2013), or cause disease. Although the utilization of soil microbes to improve sustainability of crop production is an extremely active research field, the complex nature of the interaction of microbes with plants makes it challenging to apply microbial-based solutions that harness root traits to improve soil resource capture by crops. Hence, there is growing interest in understanding root-microbe interactions.

Microbiome selection and engineering efforts are focused on selecting strains, consortia or whole communities that enhance plant growth by means of specific mechanisms. For example, nitrogen (N) fixation by diazotrophs or phosphorus (P) solubilization by soil bacteria and fungi, as well as P acquisition and translocation by mycorrhizal fungi. In parallel, plant phenotypes that may shape the composition (and functions) of root-associated microbiomes are being sought (Deng et al., 2021; Wagner, 2021). However, the plant traits that are being targeted are usually linked to interactions of root microbes with plant molecular, biochemical, and cellular traits (Connor et al., 2020), such as the composition of root exudates (Sasse et al., 2018), root metabolites (Pang et al., 2021), and plant genetic markers (Deng et al., 2021). Other approaches to disentangle mechanisms of plant control of the root microbiome involve root transcriptomic or metabolic processes using mutants. For example, mutants of *Arabidopsis* have been used to understand pathogen-defense and soil microbial recruitment under low-P stress (Castrillo et al., 2017). Also, mutants with contrasts in N use efficiency in rice were used to study microbiome recruitment and functioning (Zhang et al., 2019). Fewer research efforts have focused on the interactions of root architectural and anatomical phenotypes with root-associated microbes, although recent reports give insights into the importance of root hairs for the microbiome composition of maize (Gebauer et al., 2021), and the involvement of endodermal suberization on the recruitment of bacterial taxa using synthetic communities (Salas-González et al., 2021). Root anatomical and architectural phenotypes [and their simpler elements, or phenes – *sensu* York et al. (2013)] are being deployed as breeding targets for improved sustainable nutrient uptake and stress-tolerance by crops (Paez-Garcia et al., 2015; Koevoets et al., 2016; Wissuwa et al., 2016; Lynch, 2019), with improved tolerance of crops with shallower root systems to low-P (Burridge et al., 2019) and high salinity (Kitomi et al., 2020).

Here, we present a different perspective on the way root-microbe interactions can be viewed and understood, adding an additional dimension that has been largely unexplored, which is how phenotypic variation in root anatomy and architecture can shape diverse micro-habitats for associated microbes thereby affecting the benefits of the microbiome to the plant. Although we recognize the importance of pathogens, mycorrhizal fungi, and rhizobia in the process of nutrient acquisition by plants, we are referring here to microbes *sensu lato*, without focusing on any of these groups specifically, but providing our view on the general effects of root anatomy and architecture on the root bacterial and fungal microbiome. Adding the root phenotype to the genetic, molecular, cellular, and metabolic levels would complement our understanding of plant-microbiome interactions. We present the root system as a dynamic habitat for microbes where anatomy and architecture play an essential role in determining microenvironments for root-associated microbes and the functioning of these microbes to improve plant performance. Furthermore, we propose that microbiomes may be co-selected through plant breeding by targeting root architectural and anatomical phenotypes that favor plant growth and health, especially under abiotic stress conditions.

## Root Phenotypes as Breeding Targets to Develop Crops That Are More Efficient in Acquiring Soil Resources

**FIGURE 1:**
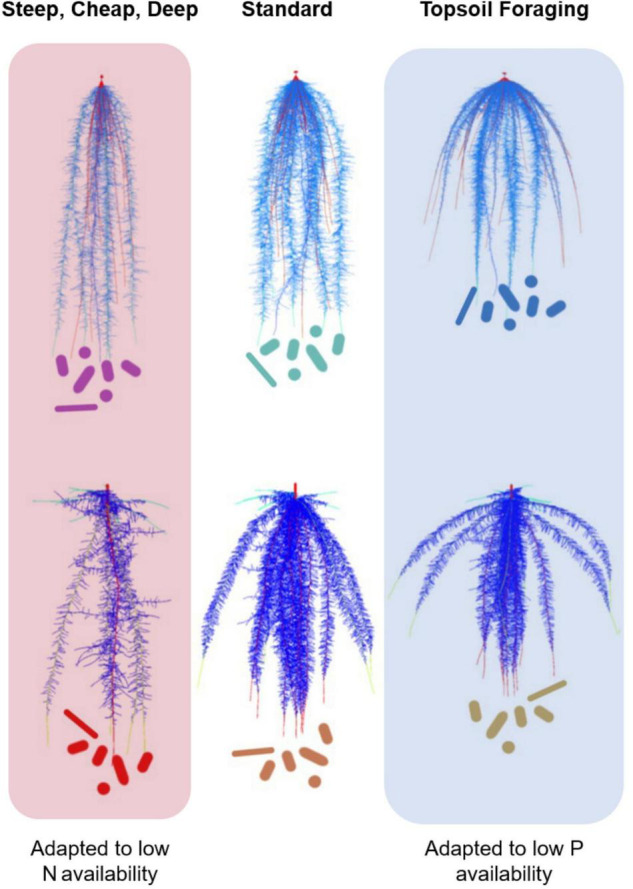
Ideotypes for maize (upper panel) and common beans (lower panel). “*Steep, cheap and deep”* root systems with, steep rooting angle, reduced metabolic cost of soil exploration, deep rooting root systems are better adapted to drought and low N availability; while “*Top soil foraging*,” root systems with shallow rooting angle, increased basal root whorl number, and increased lateral root branching have increased tolerance to low P. We propose that different root ideotypes would also have characteristic root microbiomes in the rhizosphere and endosphere, given the different niches offered by each root system. Figure modified from Lynch (2019).

Root architectural and anatomical phenotypes play primary roles in the capture of soil resources. Root architecture controls soil exploration by regulating the spatiotemporal dynamics of resource foraging in specific soil domains. Resource capture can only occur when root foraging coincides with resource availability in time and space. Root architecture directly affects resource foraging from soils by locating active roots in specific soil domains, and indirectly by regulating the metabolic cost of soil exploration (Lynch, 2019). An example of a direct effect of root architecture on soil resource capture is the growth angle of axial roots, which determines the depth of nutrient and water uptake (Figure 1). Rooting depth is critical for capturing soil resources since the bioavailability of virtually all soil resources is vertically stratified in almost all soils (Lynch and Wojciechowski, 2015). For example, P is an immobile resource whose bioavailability is concentrated in shallow soil strata, while water and nitrate are mobile resources that tend to accumulate in deeper strata (Barber, 1995). A shallow root growth angle, and other architectural phenotypes that localize root foraging in the topsoil, therefore promote P acquisition, while steeper angles improve water and N capture. An excellent example of this is the gene *DRO1*, which regulates the growth angle of nodal roots in rice (Uga et al., 2013). Rice isolines with the *DRO1* allele have nodal roots that are steeper, deeper, and therefore better able to tolerate drought stress. An example of an indirect effect of root architecture on resource capture is the number of axial and lateral roots produced. The formation of many axial and lateral roots imposes metabolic costs that can retard rooting depth and the capture of water and nitrate from the subsoil. For example, maize lines that produce many lateral roots and many nodal roots are shallower, and therefore better able to capture P, but less efficient in capturing water and N than lines with fewer root axes (Lynch, 2019, 2021).

Root anatomy also has important roles in soil resource capture, by regulating the metabolic cost of soil exploration, exploitation of the rhizosphere, penetration of hard soil domains, axial and radial transport of water and nutrients, and interactions with soil biota including endosphere and rhizosphere microorganisms (Lynch et al., 2021). An excellent example of a direct effect of root anatomy on soil resource capture is that of root hairs. Variation in root hair length and density is associated with the volume of the diffusion depletion zone surrounding roots, and therefore with the exploitation of rhizosphere resources, including most notably P (Bates and Lynch, 2000), which is highly immobile in the soil, but also including nitrate, a mobile resource (Saengwilai et al., 2021). Anatomical phenes that reduce the metabolic cost of soil exploration often improve soil resource capture (Lynch, 2015, 2019; Lynch et al., 2021). Several examples of this exist in maize, in which genotypic variation for anatomical phenotypes that alter the proportion of living tissue and cytoplasm in root tissue is associated with greater soil exploration, resource capture, and plant fitness under drought and nutrient stress. For example, maize genotypes with more root cortical aerenchyma (RCA), which replaces living cortical cells with air-filled lacunae, have less root respiration and nutrient content, increased root growth, and increased soil resource capture, resulting in greater growth and yield under suboptimal availability of water, N, and P (Fan et al., 2003; Zhu et al., 2010; Saengwilai et al., 2014; Chimungu et al., 2015b; Galindo-Castañeda et al., 2018). Similarly, maize genotypes with larger cortical cells or fewer cortical cell files have reduced root metabolic cost, greater root growth, and greater water capture, biomass, and yield under drought (Chimungu et al., 2014a,b; Chimungu et al., 2015b). In barley, root cortical senescence improves nutrient capture *in silico* by reducing living cortical tissue (Schneider et al., 2017). In dicot species, reduced secondary growth is associated with reduced metabolic cost, greater soil exploration, and better P capture under low-P stress (Strock et al., 2018). Soil exploration, especially in deep soil domains, is associated with the ability of roots to overcome mechanical impedance. Several root anatomical phenotypes are associated with penetration of hard soil, including root hairs (Bengough et al., 2016), the shape of the root tip (Colombi et al., 2017), cortical anatomy (Chimungu et al., 2015a; Vanhees et al., 2020), and multiseriate cortical sclerenchyma (Schneider et al., 2021b). Root anatomy has multiple effects on radial and axial hydraulic conductivity, which is critically important for optimizing water use throughout phenology (Vadez, 2014). As discussed below, root anatomical phenotypes could be important drivers of root and rhizosphere microbiomes, as well as other root-associated biota including nematodes and insects (Lynch et al., 2021). For example, root cortical anatomy in maize is associated with differential effects on root colonization by *Fusarium* species and beneficial mycorrhizal fungi (Galindo-Castañeda et al., 2019).

Several root phenotypes present opportunities to breed more resilient, efficient crops. As with any breeding program, breeding strategies for root phenotypes will be influenced by the genetics of the species, the phenotype of interest, and the resources available. While in many cases the genetic control of root phenotypes is complex and poorly understood, in several cases they are under simple genetic control. For example, axial root growth in rice, which is related to root depth and therefore drought tolerance, is regulated by *DRO1* (Uga et al., 2015). As another example, nodal root growth angle in maize, which is associated with root depth and N capture, is regulated by *zmCIPK15* (Schneider et al., 2021a). Such cases are amenable to genotypic selection. However, the fitness landscape of root phenotypes is often complex, being determined by interactions among multiple phenes within the integrated phenotype, as well as by interactions with the environment, and many root phenotypes exhibit fitness tradeoffs. For example, high root xylem conductance is beneficial for *Phaseolus* taxa under water stress when paired with deep root architecture, but detrimental when paired with shallow root architecture (Strock et al., 2021). Long root hairs are substantially more beneficial for P capture in common bean when paired with shallow root growth angles than when paired with deep root growth angles (Miguel et al., 2015). In maize, multiple integrated root phenotypes are optimal for drought tolerance rather than individual phene states (Klein et al., 2020). Synergism among phenes states rather than additive combinations accounted for superior N capture by rice root systems *in silico* (Ajmera et al., 2022). Many root architectural phenotypes exhibit tradeoffs between deep and shallow soil exploration (Burridge et al., 2020), which is important since some primary resources such as water and nitrate are often localized in the subsoil whereas P and other nutrients are often more available in the topsoil (Lynch and Wojciechowski, 2015; Lynch, 2019; Lynch et al., 2021). Phenotypic plasticity in response to the soil environment is an added complication, although plasticity can be considered to be a phenotype under genetic control subject to selection (Schneider and Lynch, 2020). The complexity of the fitness landscape for root phenotypes has several implications for breeding strategies. One is that it is highly improbable that brute force yield screening will identify germplasm that possesses optimal integrated root phenotypes in addition to high yield potential, disease tolerance, local adaptation, etc. This is especially true for root phenotypes, since elite germplasm, generally the product of decades of selection under high inputs, may lack the adaptations to edaphic stress found in landraces selected under low input conditions. This calls for informed phenotypic selection, i.e., *ideotype breeding* (Lynch, 2019). Direct phenotypic selection is affordable and accessible for crop breeding programs in developing countries, which generally serve production environments with more edaphic stress and limited input use, and may not have resources for genotypic selection. Field-based root phenotyping platforms, combined with the availability of inexpensive pocket microscopes, permits inexpensive, robust root phenotyping at fairly high throughput in target soil environments (e.g., Trachsel et al., 2011; Colombi et al., 2015; Burridge et al., 2016). *In silico* tools are useful for the development and evaluation of root ideotypes, since they permit the evaluation of many phenotypes in many environments, including phenotypes and environments that do not yet exist in nature, such as future climates (Marshall-Colon et al., 2017). An example of a successful root-focused breeding program is that of common bean in Mozambique (Burridge et al., 2019). Low P availability and drought are primary constraints to bean production in Mozambique, as they are in much of sub-Saharan Africa (Wortmann et al., 1998). A bean breeding program was established targeting root phenotypes improving water and P capture, focusing on basal root whorl number, basal root growth angle, and long, dense root hairs. New lines with superior root phenotypes had 2.5-fold greater yield than the best existing lines. The new lines also afforded better utilization of rock phosphate, reduced soil erosion, greater biological nitrogen fixation, acceptable competition with maize in polyculture, and greater household food security and income.

## Research Gaps in the Study of Interactions of Root Microbiomes With Root Architectural and Anatomical Phenotypes

Microbial inoculation of plants with single strains, simple consortia, entire communities (Compant et al., 2019), and soil inocula (Mueller and Sachs, 2015) have been examined as avenues to improve plant growth. Reviewing the potential of prokaryotes and fungi as plant inoculants to improve crop production is beyond the scope of this viewpoint. Here, we emphasize that most studies focusing on the effect of root-associated microbiomes on plant growth and health, overlook the effects of root architecture and anatomy on root-microbe interactions. Some studies that have addressed this gap focus on diazotrophs and study the distribution of associative bacterial species (see for example Reinhold et al., 1987) and the distribution of rhizobia nodules (for example De Cuyper et al., 2014) in root systems. Although the anatomy of the nodules or the association with other diazotrophs has been thoroughly described (Sprent, 1980; Oldroyd and Downie, 2008), no special attention has been devoted to the effects of natural variation in root anatomy or architecture across plant populations on this type of plant-microbiome association. Studies of mycorrhizae have tangentially explored root anatomy and architecture of individuals of different plant species (Dreyer et al., 2010; Sharda and Koide, 2010; Strock et al., 2018; Galindo-Castañeda et al., 2019), but mycorrhizal colonization across a wide selection of accessions of plant populations with contrasting root anatomies and architectures have, to our knowledge, not yet been studied. Descriptions of pathogen colonization where root anatomy (see references in Lynch, 2021) and architecture (see for example McPhee, 2005 and references cited by them) have been phenotyped are scarce, therefore lacking the perspective of the effect of these root traits on pathogen colonization. Some studies have, however, investigated the genetic control of pathogen resistance and have linked this with genetic markers of root architecture (for example Hagerty et al., 2015). Comprehensive microbiome surveys have rarely focused on root anatomy and architecture, but the interest in this topic is rapidly growing, especially from the ecological perspective. The functional significance of root traits in natural plant populations, and the application of those findings in agriculture is gaining more attention (Abalos et al., 2019).

Although valuable and relevant, recent research about root traits and associated microbiomes in *Arabidopsis* (Fröschel et al., 2021; Salas-González et al., 2021), *Brachypodium* (Kawasaki et al., 2016), maize (Gebauer et al., 2021; Rüger et al., 2021), wheat and rice (Kawasaki et al., 2021) has been performed using pots where root anatomy and architecture can barely be expressed or measured. In the experimental systems used by the mentioned studies, gradients of different nutrients and environmental conditions were not considered (either because pot size was too small, or because it was not part of the research question), and horizontal gradients of root exudates might not be realistic given the extent to which they diffuse from the rhizosphere into the bulk soil. Furthermore, field studies have focused on the genetic control of plant-microbe interactions (Peiffer et al., 2013; Deng et al., 2021), while observations on the interaction of root anatomical and architectural traits with microbial associations are still rare at the field scale and mostly focused on the root tip and adjacent zones (Yu et al., 2018, 2021; Zai et al., 2021). Nevertheless, some insights into rooting depth and branching order of trees (King et al., 2021; Zai et al., 2021) and root class effects on the microbiome in *Nicotiana tabacum* (Saleem et al., 2016) have been made.

Plants selected for improved soil resource capture might sustain specific microbial taxa depending on the quantity and quality of root exudates and the physicochemical changes induced by root architectural and anatomical phenotypes that have adaptive value for the capture of scarce resources. Moreover, there are many cases in which abiotic stresses has been associated with an increase in carbon rhizodeposition, through increased exudation (e.g., low P and excess aluminum), root loss (for example under drought), and increased biomass allocation belowground (e.g., low P, low N, and drought), as reviewed by Neumann (2007). However, plant-centric experimental evidence indicates that efficient use of carbon by the plant is made through reduced allocation of carbon to build metabolically “cheaper” root tissues. This reduction of metabolic cost of soil exploration has been proposed and has received a wealth of experimental support (Lynch, 2018, 2019). Therefore, studies focusing on root microbiomes should explore root environments where carbon allocation might be reduced in order to explore such limiting soil environments under abiotic stress. How the plant balances its carbon economy to sustain a root system that has a metabolically reduced cost, while also sustaining a beneficial microbiome is a question that merits attention.

## Root Phenotypes Are Niche Modifiers of Root-Associated Microbes

**FIGURE 2:**
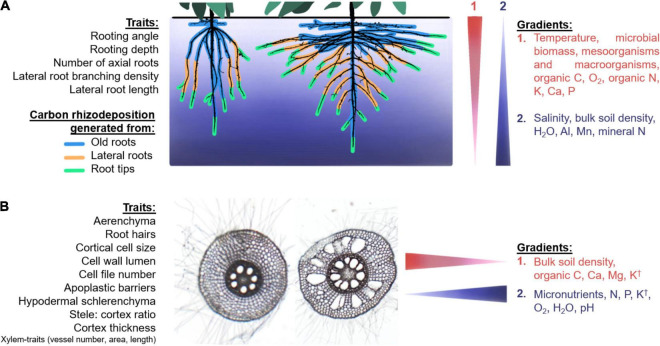
Hypothesized modifications of microhabitats in the endosphere and rhizosphere caused by vertical **(A)** and horizontal **(B)** soil gradients, shown as narrow triangles (on right) to indicate the direction of the gradient. **(A)** Two common-bean root systems with contrasting root architectures (modified from two actual root images courtesy of Dr. Magalhaes A. Miguel). The colors around the roots represent different origins of carbon rhizodeposition, with old roots shedding tissue from secondary growth and root exudates. Deep rooting systems deposit different composition of exudates in deeper soil layers due to the increase in root tips in deeper locations. **(B)** Two root cross-sections of maize taken in the same spot within the root crown in two different plants showing differences in root anatomy. Anatomical phenotypes and environmental gradients that we hypothesize to be related to microbial associations in the rhizosphere or endosphere are listed in regular font size, although indirect effects of the phenotypes listed in small-sized font with the gradients may also be expected. **†** K effect can vary between different sites with different soil physicochemical conditions. Details on the specific root phenotypes and their interactions with microbes are provided in the text.

Root architecture (e.g., rooting depth, root growth angle, lateral root branching density, number and class of axial roots) and anatomy (e.g., root cortical senescence, RCA, and root secondary growth, production of apoplastic barriers, root hair length and density) determine the structural characteristics of niches and microhabitats for root-associated microbes. The fitness landscape of root phenotypes, still to be explored and fully understood, offers many diverse possible combinations of root phenes and phene-states, resulting in different root ideotypes for plant growth under specific environmental constraints (Rangarajan et al., 2018; Klein et al., 2020). Each of these combinations or integrated root phenotypes correspond to a point in the fitness landscape for microbial associations as well. Furthermore, the interaction of architecture and anatomy with soil properties and spatiotemporal gradients might offer an additional range of possibilities of niches and habitats for soil microbes recruited into the rhizosphere and endosphere (Figure 2).

In the next paragraphs we describe examples of possible (and hypothetical) niche modifications determined by root architecture and anatomy that may affect root-associated microbes, as well as their interactions with the soil vertical and horizontal gradients, focusing on suboptimal nutrient availability. We do not attempt to explore the effects of microbes on root anatomy and architecture, but we acknowledge that microbial feedbacks affecting root phenotypes exist (Saikkonen et al., 2020; Salas-González et al., 2021), as observed with other organisms, such as insects (Gould et al., 2018). Although the mechanisms of associations between plants and pathogenic, mycorrhizal, or symbiotic diazotrophs are well characterized (as referenced in the previous section), the mechanisms of associations with other microorganisms, especially those with plant-growth promoting characteristics, remain elusive.

### Root Architecture

Root architectural phenotypes such as rooting angle and depth, number of axial roots, lateral root branching density and lateral root length might be important factors determining composition and function of root microbes. It is logical that deep-rooting plants would allocate more carbon rhizodeposition to deeper soil layers given that root caps and border cells, mucilage and root exudates are produced in larger amounts (although not uniquely for root exudates) at the root tips and root elongation zone (Badri and Vivanco, 2009; Jones et al., 2009; Massalha et al., 2017). Our hypothesized changes in root-derived C allocation with two contrasting root architectures are depicted in Figure 2A. In general, C of deep-rooting plants is expected to be allocated deeper in the soil profile. Accordingly, microbial interactions due to physicochemical and biological gradients are expected to be different in deep-rooted phenotypes compared to shallow-rooting plants. Plants with increased axial root numbers might cause steeper gradients in the nutrient depletion zones, especially in the epipedon, which could affect the microbial communities utilizing these resources in the bulk soil. It is possible that plants with more axial roots and increased lateral root density and lateral root length may host a greater microbial biomass due to more root tips and the resulting microbial feeding sites. More axial roots may also lead to an increased number of lateral root junctions, through which bacteria can enter the root cortex (Liu et al., 2006). In contrast, roots with reduced lateral root density would display reduced attachment surfaces for microbes, reduced entry points for endophytes, and reduced carbon rhizodeposition, which could cause a decreased capacity to sustain microbial communities. For example, genes involved in initiation and emergence of lateral roots (in addition to root hair length and root morphogenesis) in field-grown *Arabidopsis thaliana* are linked to the microbial composition of the rhizosphere (Bergelson et al., 2019), which is in agreement with our proposed statements. The fact that lateral roots, compared to axial roots, sustain a different community in *Nicotiana tabacum* (Saleem et al., 2016), as well as the finding that specific root length was the most important root characteristic explaining differences in rhizobacterial community composition of green-house grown *Phaseolus vulgaris* (Pérez-Jaramillo et al., 2017), support our proposal that the selection for root architectural phenotypes results in the concomitant selection of the associated microbiomes.

Indirect effects of root architecture on soil microbial communities within the time frame of a crop season (e.g., not considering long term effects) might be related to the rate of root decay and its links with soil organic matter priming. The priming effect, i.e., accelerated decomposition of soil organic matter following fresh organic matter input to soil, is often supposed to result from a global increase in microbial activity due to the greater availability of energy released from the decomposition of fresh organic matter (Kuzyakov et al., 2000). Soil organic matter priming could be caused by the release of root exudates and/or fine roots into the soil (Swinnen et al., 1995). Root decay early in the season might have different effects on the rhizosphere microbial communities of plants with decreased axial or lateral root densities compared with plants with greater root density. Additionally, plants with greater lateral root densities closer to the soil surface may increase decaying organic material later in the season to spike the release of nutrients that might be readily available for plant uptake, compared to plants with reduced lateral root densities.

This priming of soil organic matter decomposition will be even more pronounced at depth because deeper soil layers have less microbial activity due to less resource input, reduced oxygen availability, and greater moisture content. However, when roots do reach deeper, they (1) introduce more rhizodeposition that contains primary resources (e.g., C and N) for microbes, (2) reduce moisture content due to water uptake, and (3) when roots turn over, the remaining macropores enhance oxygen diffusion and water infiltration. These changes in soil properties and processes can lead to a priming of soil organic matter (Fontaine et al., 2007), especially in the short-term. In the long-term, however, the increased carbon input through root decay, together with the improved soil structure due to increased microbial activity and biopores, will most likely lead to an accumulation of soil organic matter and thus resources for the plant.

### Root Anatomy

**FIGURE 3:**
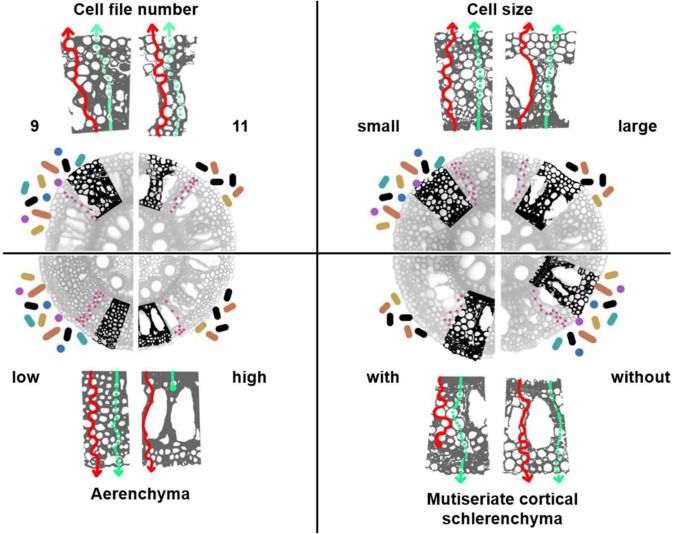
Hypothesized rhizosphere and endosphere microbiome interactions with the root anatomy of a cereal. Images of root cross-sections of maize axial roots and detail (bolded excerpt) of the expected changes in root exudation from the apoplastic (red solid arrows) or symplastic pathway (green dashed arrows). Four phenotypes are depicted: cell file number (9 vs. 11), which indicates the number of concentric cell layers in the cortex; RCA (low vs. high), the production of air pockets in the cortical tissue; cell size (small vs. large); MCS (with vs. without), lignified schlerenchyma in the peripheral cortical tissue. We propose that each of these phenotypes has an associated pattern of root exudation that may affect rhizosphere (icons indicated with colored circles and bastons around the roots) and endosphere (pink circles in the root cortex) microbial communities. Although not to real scale, the quantity of the microbe icons indicates our proposed hypotheses on microbial abundance. Colors in the rhizososphere microbe-icons indicate our proposed hypothesis about effect of root anatomy on biodiversity, with more colors indicating increased species or functional diversity.

Root anatomy is directly linked to adaptation to abiotic and biotic stress in crop species and therefore might be linked to the composition and functions of the root microbiome. Traits such as root diameter (and related traits like cortical cell number and size), RCA, the production of apoplastic barriers in the cortical tissue (Figure 3), and secondary growth in dicots (Strock and Lynch, 2020) might be important for root-associated microorganisms. In a recent study coupling genome-wide association studies (GWAS) with root microbiomes of with field-grown *Arabidopsis* (Bergelson et al., 2019), the authors report that one of the categories of genes with the highest association indexes to the presence of bacteria belong to root development (radial pattern formation and root morphogenesis), which supports the idea that root anatomy is an important factor linked to root microbial composition. Additionally, root anatomy is related to root carbon exudation (Canarini et al., 2019), an important component of carbon rhizodeposition. Root exudation has been in general considered a process driven by diffusion, but active transporters of primary and secondary metabolites in the cortex of several plant species suggests the involvement of the symplastic pathway (Sasse et al., 2018). If targeting specific root traits is an aim of plant breeding, the interactions between these traits and carbon rhizodeposition as well as radial physicochemical gradients in roots must be considered. In the next paragraphs we provide detailed hypotheses regarding possible interactions of root anatomical phenotypes and microbial colonization.

Root diameter can be understood as a phene aggregate (York et al., 2013) composed of a subset of anatomical traits that might be linked with microbial associations. We propose that the thickness and biochemical composition of the outermost layer of each root, may constitute a barrier for the release of root exudates that diffuse through the apoplastic spaces when lignified or suberized, especially in axial roots or lateral roots of first orders. Changes in the rate of exudate diffusion as well as in the chemical composition of the shed tissue as roots grow (resulting for example from secondary growth in dicots, or root cortical senescence in monocots), may affect the microbiome that feeds on these carbon compounds (Strock et al., 2018). Also, hypothetically, roots with a thicker epidermis or hypodermis will probably have reduced microbial abundance and diversity in the rhizosphere, and perhaps this may also affect the recruitment of soil-born microorganisms in the endosphere toward a less abundant and diverse microbial community. However, open spaces in the epidermis, a product of the intercellular spaces in the epidermal cells, have been described as hotspots of microbial activity [reviewed by Jones et al. (2009)], suggesting that even thick, old, lignified or suberized roots may sustain a microbial community with different composition and biomass than thin, young roots. Nevertheless, increased surface area and longer times of consolidation for the microbial communities of thicker roots compared to young and thineer roots, may also offer an opportunity for microbes to establish and flourish in thick, lignified roots. These hypotheses require further investigation.

Cortical thickness is phene aggregate that comprises the traits number of cell files, cell size and cell number. Cortical thickness may affect microbial associations in the endosphere because the associated traits determine the total apoplastic space and the length of the symplastic pathway through which the exudates travel to reach the rhizosphere. In addition, the apoplast contains the space where hyphae extend (in the case of fungal associations), and where other endophytic organisms establish in the cortex. Roots with increased apoplastic volume such as roots with augmented cortical cell numbers and decreased cell sizes, with a more complex apoplastic pathways, may harness a more diverse and abundant microbial community compared to plants with reduced cell numbers and bigger cells, which may sustain a simpler and smaller apoplastic volume (Figure 3) (Galindo-Castañeda et al., 2019). The existence of natural variation in the cortical thickness in monocots (Schneider et al., 2020) and the variation in secondary growth in dicots (Strock and Lynch, 2020) might therefore be correlated with contrasting root microbiomes for plants from the same plant species but that have different root diameters. Experimental evidence supports this hypothesis; for example, root rot severity of field-grown maize was directly correlated with cell file number and inversely correlated with cell size in different hybrid lines; which was explained as variation of apoplastic space in the cortex (Galindo-Castañeda, 2018; Galindo-Castañeda et al., 2019). In the same study, root colonization of *Fusarium verticilliodes* in greenhouse-grown inbred maize was reduced in lines with greater root cortical area. Additionally, greater root diameters associated with reduced bacterial diversity in the tree *Robinia pseudoacacia* (Zai et al., 2021), and similarly, lateral roots of *Nicotiana tobacco* (Saleem et al., 2016) and fine root branching orders of *Populus* × *euramericana* (Wang et al., 2017) had increased bacterial diversity compared to thicker, axial roots. The study by Pérez-Jaramillo et al. (2017) showing effects of specific root length on microbiome composition suggests a relationship between root anatomy and recruitment of microbes, as this trait is as strongly influenced by root diameter (and other cortical traits) as it is by root length. However, the effects of anatomical traits associated with root diameter and root class on the microbial communities are still unknown for most crop species.

The presence of apoplastic barriers, deposits of suberin in the intracellular spaces of the cortex, across the different cell layers from cortex to stele, is known to reduce the passage of microorganisms, especially pathogens (Schreiber et al., 1999). Apoplastic barrier thickness and degree of suberization is regulated by plant hormones and triggered by environmental cues; for example, endodermal thickness and its degree of lignification or suberization is enhanced when plants are under stress (Enstone et al., 2002). Additionally, rice lines with increased suberization of the endodermis withstand drought better (Henry et al., 2012), possibly by reducing water lost to the cortex or rhizosphere. Precocious suberization of the endodermis has been linked to pathogen resistance (Garrett, 1981); therefore, endodermal thickness due to increased suberization is an anatomical trait that might be important in the establishment of microbial associations. The composition of the rhizosphere and endosphere microbiome of *Arabidopsis thaliana* is correlated with the regulatory network controlling the endodermal suberization of the roots system, showing bacterial species-specific degree of suberization in inoculation experiments (Salas-González et al., 2021). The effects and coordination of interaction between the root microbiome and endodermis in crop species is, however, still unknown.

Suberization and lignification of hypodermal cells is also a possible variation in root anatomy that could be related to microbial colonization (Schreiber et al., 1999). The presence of lignified and thickened cells in the root cortex of cereals, multiseriate cortical sclerenchyma (MCS), was recently shown to be correlated with tolerance of maize and wheat to soil compaction (Schneider et al., 2021b). Given that lignified cells may restrict passage of solutes, organic compounds, and microorganisms between the inner cell layers in the cortex and the adjacent cells, we propose that the degree to which this phenotype is expressed might be correlated to microbial colonization in the axial roots of cereals (Figure 3). In plants with this phenotype, exudate secretion as well as cell shedding might be reduced, leading to a less diverse and less abundant microbial community in the rhizosphere. Additionally, the colonization of endophytes entering the root *via* rhizosphere in roots with MCS might be reduced.

**FIGURE 4:**
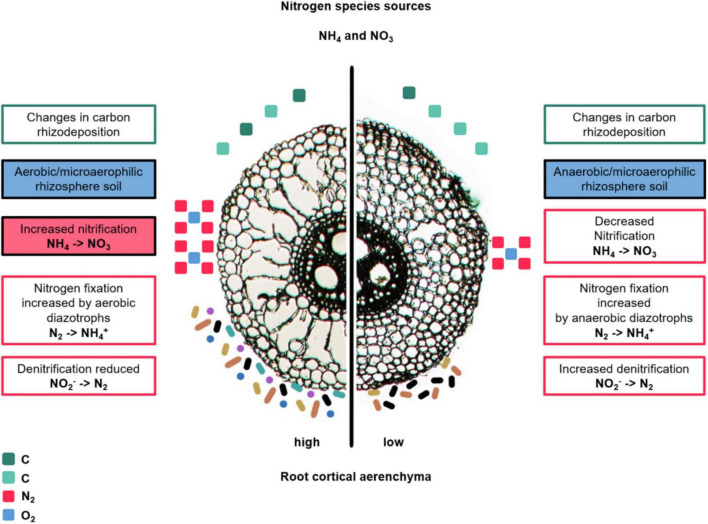
Hypotheses about changes in diversity, abundance and N transformation capacity of the bacterial community in the rhizosphere of maize due to changes in RCA. The text boxes describe hypotheses about root-derived carbon, air, and nitrogen transformation. Carbon rhizodeposition is represented by green square icons; the number of icons is related to the hypothesized change in amount of C deposited at each RCA phenotype. The more icons, the more carbon. The differences in green tones indicate changes in biochemical composition. Air is represented by blue and pink square icons, corresponding to O_2_ and N_2_, respectively. The number of icons represent the hypothesize change in amount of air. The colored bastons and circle icons represent bacterial communities and their different colors represent diversity (more colors, greater diversity); the number represent the expected microbial abundance (not to scale or proportional to the actual amounts). The hypothesis for which experimental support exists are in color-filled text boxes (Risgaard-Petersen and Jensen, 1997; Arth and Frenzel, 2000; Kennedy et al., 2004; Li et al., 2008; Galindo-Castañeda, 2018), while hypothetical statements are written in non-color-filled text boxes.

The production of RCA has major impacts on the rhizosphere by changing oxygen availability, thereby reducing abundance and activity of anaerobic bacteria and archaea, and favoring microorganism with aerobic metabolisms in the vicinities of RCA air pockets (Risgaard-Petersen and Jensen, 1997; Arth and Frenzel, 2000; Li et al., 2008) (Figure 4). Processes like microbial carbon utilization, N transformation, and metal accumulation in soil depend on oxygen concentrations and redox potentials (Neumann and Römheld, 2012), thus potentially are susceptible to changes due to RCA. For example, when rice shoots were clipped and O_2_ and N_2_ were no longer transported to the rhizosphere by means of RCA channels, nitrification and denitrification were significantly decreased in planted rice soils (Arth and Frenzel, 2000). A rice genotype producing greater RCA had increased nitrification activity, nitrate concentration and abundance of ammonia oxidizing bacteria in the rhizosphere compared to a genotype with decreased RCA formation (Li et al., 2008). These results were in accordance with the stimulatory effects of aerenchymatous roots of the aquatic plant *Lobelia dortmanna* on rhizosphere nitrification (Risgaard-Petersen and Jensen, 1997). Conversely, roots with reduced RCA may restrict oxygen diffusion to the rhizosphere, thereby limiting aerobic microbial metabolism and nutrient utilization. When maize inbreds were studied for their rhizosphere prokaryotic communities in relation with RCA formation under low-N stress, specific taxa of the families Burkholderiaceae and Bacillaceae were enriched in low-RCA plants compared to high-RCA plants (Galindo-Castañeda, 2018). The authors hypothesized that plants with low-RCA may be able to sustain such enrichments due to increased root exudation specifically targeted to these bacterial families. Root exudation of contrasting-RCA plants has not been studied to our knowledge, but we hypothesize that increased aerenchyma restrict the transport of exudates in axial roots due to the reduction of the symplastic and apoplastic transport pathways in the cortex (Figure 4). The effect of RCA in lateral root exudation has not been studied, but merits attention given that lateral roots have the greater microbial abundance and diversity, compared to axial roots, as noted in the section above (Root architecture).

To summarize our hypotheses regarding root anatomy and microbial interactions, contrasting root anatomical phenotypes might have an associated pattern of root exudation, area for microbial attachment, or space to host microbes. Reduced exudation and therefore, less abundant and diverse microbial communities may be found in roots with increased cell size, increased number of cell files, increased RCA, and presence of MSC. Accordingly, roots with the opposite phene states (e.g., smaller cell size, reduced number of cell files, reduced RCA, and absence of MCS) would be expected to have more diverse and abundant microbial communities in the rhizosphere. Furthermore, we propose that anatomical phenotypes determine the apoplastic space where endospheric microbial communities inhabit (represented as purple dots in Figure 3). Roots with reduced cell files, augmented RCA, increased cell sizes, and lacking MSC would have a smaller apoplastic space in which these microbes could colonize, reducing their diversity and abundance. The linkage between spatial and temporal variation in root anatomy and architecture with other root phenes at the cellular and molecular level (for example, exudates, carbon rhizodeposition, metabolite concentration, secretion of other compounds) is poorly understood and merits attention. In addition, understanding the spatial-temporal organization of root anatomy and architecture in the context of cellular and biochemical processes is crucial for understanding root-microbial associations.

## Synchronizing Root and Microbiome Engineering

**FIGURE 5:**
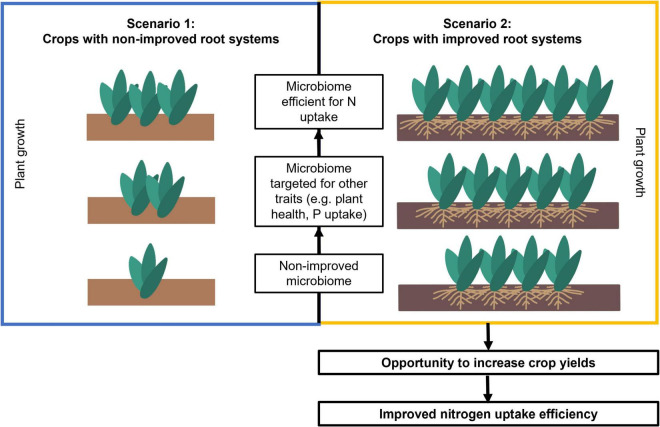
Scenarios for root-microbiome engineering with and without including plant-breeding targeting root traits, using the example of N uptake as the most important abiotic stress in the location where breeding is occurring. The focus of the target in the plant breeding part have been detailed (only shoots in scenario 1, shoots and roots in scenario 2). We hypothesize the greatest margin of improvement might be obtained when root breeding and microbiome breeding are combined (shown here as the plant drawing with the most numerous individuals) compared to a basic margin of improvement with only one plant when neither of these two approaches are considered. Within each of the scenarios, narrowing the stressor factors to the most important one at the local level (low nitrogen in the example), is hypothesized to provide the best yields. The black arrows indicate the expected direction of yield increase if the different approaches are taken. It is assumed that root microbiomes have the potential to improve plant growth when applied in the field, but this assumption is still a matter of research and technological innovation.

It is possible to design and modify the root microbiome to promote resource capture by plants, plant stress tolerance, and phytohormone production – as reviewed by Orozco-Mosqueda et al. (2018). However, few studies have explored the possibility to engineering the root microbiome in parallel with the selection of root anatomical and architectural phenotypes. In fact, few studies have addressed both root adaptation and microbiome selection to enhance plant response to abiotic stress (Castrillo et al., 2017; Salas-González et al., 2021; Yu et al., 2021). We propose that optimal combinations of root phenotypes (or ideotypes) and beneficial microbiomes can be matched and engineered together to improve plant growth under specific stress conditions (for example under low-nutrient or drought). This new perspective aims at broadening the margins of plant yield improvements by searching for synergies in which benefits of selected root traits and root microbes might be mutually enhanced (Figure 5). According to this perspective yield increase can be improved using two possible scenarios as follows. Scenario one is not focused on root phenotypes to improve soil resource acquisition, and rather focuses on engineering root microbiomes in combination with existing crop breeding programs that focus on yield and shoot phenotypes (Figure 5, scenario 1). In scenario two, root phenotypes are engineered together with their associated microbiome to obtain the maximum yield improvement (Figure 5, scenario 2).

Improvements within each of these scenarios could be further reached if environmental constraints are considered for the selection of roots and associated microbes. We propose that by breeding plants and microbes under abiotic and biotic stresses that are common in crops such as drought, pathogens, soil compaction, and reduced nitrogen or phosphate availability, the resulting plant-microbe associations would perform better compared to those selected under optimal growth conditions. Further improvements could be expected if site-specific stress factors are used for the selection and breeding. For example, low nitrogen could be a constraint in low-input agroecosystems, but not in high-input agroecosystems. Therefore, selecting microbiomes and roots under low N would work better for low-input agroecosystems than for high-input ones. Accordingly, selection stressors for European agroecosystems should be done under, for example, soil compaction to get the greatest yield increase in Europe.

## What Is Needed to Develop Synergies Between Root Traits and Microbiomes?

In order to develop the proposed perspective, we present research gaps that could be addressed as ways to facilitate our understanding of the interactions of root microbes and plant root architectural and anatomical phenotypes:

- Experimental systems that enable analysis of both root phenotypes and microbiomes in the same plant. Root architecture cannot be studied in small pots. The anatomical and architectural responses of roots to stress are not fully developed in small pots. Additionally, experimental systems where actual field soil containing a native microbiome, rather than potting mixtures, should be preferred. Alternatives could include inoculation of sterilized soils with native inoculants.

- Studies that test simple hypotheses about single trait effects on microbial associations would help advance our understanding. We provide a list of root phenotypes that have been or are being studied and their potential effects on microbial associations and possible related mechanisms (Supplementary Table 1).

- During the proof-of-concept stage of new root inoculants to improve plant productivity, researchers should be more aware of root system architecture and the possible interactions with soil gradients when reporting microbiome composition and functions. Informing the sampling site and a reference to root architecture would help to better understand the effects of root phenotypes on microbial associations. There are some advances in recently reported microbiome surveys of crops, such as the work of Vescio et al. (2021), describing differences between seminal and crown root microbiomes in greenhouse-grown maize, and the work by Yu et al. (2021) informing the location of their sampling points in field-grown maize. Research done with trees (Pervaiz et al., 2020; King et al., 2021; Zai et al., 2021) surveying microbes per root order show the differences that exist in microbiome composition and abundance along the different root zones and orders.

- In order to quantify root anatomical or architectural effects on plant growth, and to compare this with microbial effects, plants with contrasting root phenotypes are needed. The use of germplasm that can be tested and that has fairly stable root architecture and anatomy would help in this regard (i.e., reduced plasticity to varying environmental factors). The use of genetically modified lines has shown potential to understand genetic control of root-microbe interactions. For example, lines lacking the production of root hairs or lateral roots, are available for maize (Paszkowski and Boller, 2002; Ganther et al., 2021); or mutants with modifications in the root endodermis suberization and Casparian strip have been used in *Arabidopsis* (Salas-González et al., 2021). Nevertheless, mutants are extreme phenotypes with possible pleiotropic effects that do not represent the natural phenotypic variation present in plant populations. This makes it challenging to extrapolate results obtained with mutants to actual effects of varying phene states and their functional relevance for the plant. Therefore, more studies on the genetic control of root traits of crops and other important crop species are needed to either generate genetically modified plants, or to select plants with contrasting specific root architectural and anatomical traits that are still unexplored.

- Linked to the previous point, we need experiments determining the hierarchy of events that lead to plant growth promotion. Are roots the determinant of microbes that then enhance resource uptake in soils, or are microbes first modifying root architecture or anatomy to produce the plant growth promoting effect? Can these interactions be controlled?

- Including microbial interactions in architectural/structural models of plant growth will help advance our understanding of these interactions. While there are models that involve microorganisms and nutrient pools as they are affected by plants in soils [for example, Fatichi et al. (2019)], to the best of our knowledge, there are no structural models of plant growth that include microbial processes and nutrient capture. A better understanding of the interaction of root architecture and anatomy with microbes would help in including the interaction in functional-structural models of plant growth.

- We need a better understanding of the utility of individual root phenes and root phene aggregates on plant performance under stress in order to test our hypothesis about their interactions with microbes.

- More exploratory research is needed to understand the distribution of exudates along root systems and across different root classes. The work by Kawasaki et al. (2016) makes a comprehensive description of root exudates and root microbiome along the root system of *Brachypodium distachyon*; similar studies are needed for other crops and that can be performed in mesocosms and under field conditions. Pot experiments using small soil volumes that are a fraction of the actual soil volume explored by the plants in the field are not suitable to study root anatomy and architecture. However, the use of mesocosms that resemble the soil volume explored by the plant in the field could be a good tool for this proposed research. For example, 30 L mesocosm for maize plants of up to 4 weeks old is a good approximation of the soil volumes that the plants explore under field conditions (Galindo-Castañeda et al., 2018).

- Studies of the interaction of soil physicochemical gradients with root colonization would also help to understand the relative utility of microbes and root traits in plant tolerance to soil stress. For example, the effect of soil compaction on microbial root colonization would be an interesting topic to complement root studies. Specifically for soil compaction, we hypothesize that oxygen limitation and altered physical pore space would also change microbial communities recruited to the root surface, as it happens with bulk soil (Longepierre et al., 2021).

## Conclusion

Here we present our view of the root system as a habitat for microbes at the anatomical and architectural levels of organization, and how intraspecific variation in root phenotypes could lead to diversity in microbial niches that can be selected through plant breeding. We have identified research gaps comprising root phenomics that will complement our current understanding of root microbial associations. Building on these two until now independent research fields, and bridging root phenotyping with root microbiology for developing a more resource-efficient and stress-resilient agriculture, we expect to draw the attention of the scientific community toward novel, integrative research questions that might be tackled in the coming years.

## Data Availability Statement

The original contributions presented in the study are included in the article/Supplementary Material, further inquiries can be directed to the corresponding author.

## Author Contributions

TG-C and JPL conceived the idea. All coauthors contributed equally to hypotheses formulation, prospective applications, figures, and writing of the manuscript.

## Conflict of Interest

The authors declare that the research was conducted in the absence of any commercial or financial relationships that could be construed as a potential conflict of interest. The handling editor declared a past collaboration with several of the authors JS and MH.

## Publisher’s Note

All claims expressed in this article are solely those of the authors and do not necessarily represent those of their affiliated organizations, or those of the publisher, the editors and the reviewers. Any product that may be evaluated in this article, or claim that may be made by its manufacturer, is not guaranteed or endorsed by the publisher.
