# Supplementary material for: Improving Soil Resource Uptake by Plants Through Capitalizing on Synergies Between Root Architecture and Anatomy and Root-Associated Microorganisms
Source: Front Plant Sci. 2022 Mar 9;13:827369. doi: 10.3389/fpls.2022.827369 (PMC8959776; doi:10.3389/fpls.2022.827369)
Supplement: Supplementary file 1 [file Table_1.pdf]

**Supplementary table 1.** Root anatomical and architectural traits with their states and the hypothesized effect on exudate production and associated microbiome.

| Organizational level | Root trait | Trait states | Hypothesized effect on root exudation, physicochemical conditions, and habitat space for microbes | Hypothesized effect on root microbiome | Supporting evidence (R: Rhizosphere, E: Endosphere) |
|---|---|---|---|---|---|
| Anatomy | Root diameter | Thicker vs Thinner | • Increase in surface area per root length for microbe attachment and root exudation.<br>• Reduced total amounts of exudates per root mass or length unit.<br>• Reduced and/or different composition of exudates.<br>• Increased amount of shedding tissue per root length unit. | • Decreased diversity in the rhizosphere.<br>• Increased diversity in the endosphere. | **R** : (Saleem et al., 2016; Wang et al., 2017; Galindo-Castañeda et al., 2019; Zai et al., 2021)<br>**R and E**: (Zai et al., 2021) |
| | Cortical cell size | Large vs Small | • Reduced apoplastic and symplastic pathway length for the exudates to be released into the rhizosphere.<br>• Reduced intercellular space and horizontal environmental gradients in the cortex for microbial diversification | • Reduced diversity and abundance in the endosphere and rhizosphere. | Not reported in literature |
| | RCA or cortical senescence | Augmented vs Reduced | • Reduced apoplastic and symplastic transport of exudates into the rhizosphere.<br>• Increase of $N_2$ and $O_2$ concentration in the endosphere.<br>• Reduced cellular space for microbial colonization but possible channels for spread of microbes along the root axis. | • Changes (likely reduction) in diversity and abundance in the endosphere.<br>• Selection favoring aerobic metabolism in both, endosphere and rhizosphere.<br>• Possibly, C-degraders in the endosphere during RCA formation. | **R:** (Risgaard-Petersen and Jensen, 1997; Arth and Frenzel, 2000; Li et al., 2008; Galindo-Castañeda, 2018)<br>**E:** (Galindo-Castañeda et al., 2019) |
| | Apoplastic barriers and lignified layers in the hypodermis | Present vs Absent | • Reduced amounts of exudates transported to the rhizosphere.<br>• Barriers for microbial colonization in the cortex or selection towards organisms with the capability to degrade these barriers (likely pathogens). | • Reduced diversity and abundance in rhizosphere and endosphere.<br>• For barriers in the epidermis vs barriers in the hypodermis, differences might be expected. | **E:** (Fröschel et al., 2021)<br>**R and E:** (Salas-González et al., 2021) |
| | Root hair density and length | Long, dense vs Short, scarce | • Greater amounts of exudates in the rhizosphere.<br>• Extended surface for microbial attachment.<br>• Steeper gradients in nutrients in the rhizosphere due to increase nutrient uptake by root hairs, favoring microbial diversification. | • Increased diversity and abundance in the rhizosphere, similarly in the endosphere due to increase microbial attachment and entrance points on the root surface. | **R:** (Robertson-Albertyn et al., 2017; Holz et al., 2018; Burak et al., 2021; Gebauer et al., 2021)<br>**E:** (Schweiger et al., 1995; Brown et al., 2013) |

| | | | | | |
|---|---|---|---|---|---|
| | Secondary growth | Reduced vs enhanced | • Increased exudation rate.<br>• Reduced cortical space for endosphere microbes.<br>• Tissue shedding reduction in the rhizosphere | • Changes in biodiversity and microbial associations in the rhizosphere, although it is not clear what would be more important between increased carbon exudation and decreased tissue shedding to determine the rhizosphere microbial diversity and abundance.<br>• Likely reductions in the biodiversity and abundance of microbes in the endosphere. | **E:** (Strock et al., 2018)<br>**R:** Some hypothesis proposed by (Strock and Lynch, 2020) |
| Architecture | Rooting angle and depth | Steep – deep roots vs shallow – shallow roots | • Increase in percentage of fixed carbon allocated to deeper soil layers.<br>• Increase of microaerophilic or anaerobic pockets around the rhizosphere of root tips.<br>• Soil compaction affecting the rhizosphere. (see Figure 2) | • Associations with microbes tolerant to hypoxia, reduced rates of microbial consumption of root-derived carbon.<br>• Reduced predation by protozoa. N fixation may be favored in the rhizosphere of plants containing RCA. (see Figure 1) | Not reported in literature |
| | Number of axial roots | Many vs few | • Increased amounts of root exudates.<br>• Increases surface for microbial attachment.<br>• Larger amounts of substrate to be degraded from root decay<br>• Steeper gradients of nutrient concentration in the depletion zone in the horizontal axis. | • Overall increase in microbial abundance and diversity.<br>• Diversification in metabolism under more pronounced nutrient and water gradients in the depletion zones around the roots.<br>• Endophytic colonization increased. | Not reported in literature |
| | Lateral root density | High vs Low | • Greater number of exudation points from lateral roots.<br>• Greater number of attachment points for microbes.<br>• Greater number of entry points for endophytes in the emergence point of each lateral root. | • Overall increase in microbial abundance and diversity.<br>• Diversification in metabolism under more pronounced nutrient and water gradients in the depletion zones around the lateral roots.<br>• Endophytic colonization increased. | **E:** (James et al., 1994)<br>**R:** (Schmidt et al., 2018) |
| | Lateral root length | Long vs Short | • Root exudates allocated farther from the axial root, or from roots of lower-branching orders. Overall increase of exudates per plant. | • Differences in composition of the lateral root microbiome given the different horizontal gradients of resources between the two phenotypes.<br>• Longer roots may recruit a more diverse microbiome due to the increased soil volume explored.<br>• Lateral-root associated microbes might be facing reduced environmental stress given the decreased resource gradients as the distance from the axial roots increases. | Not reported in literature. Studies on lateral root vs axial root:<br>**R:** (Saleem et al., 2016)<br>**R and E:** (Zai et al., 2021) |

## References cited in the supplementary table that are not cited in the main text


Brown, L.K., George, T.S., Barrett, G.E., Hubbard, S.F., and White, P.J. (2013). Interactions between root hair length and arbuscular mycorrhizal colonisation in phosphorus deficient barley (*Hordeum vulgare*). *Plant Soil* 372**,** 195-205. doi: 10.1007/s11104-013-1718-9.

Burak, E., Quinton, J.N., and Dodd, I.C. (2021). Root hairs are the most important root trait for rhizosheath formation of barley (*Hordeum vulgare*), maize (*Zea mays*) and *Lotus japonicus* (Gifu). *Ann. Bot.* 128**,** 45–57. doi: 10.1093/aob/mcab029.

Holz, M., Zarebanadkouki, M., Kuzyakov, Y., Pausch, J., and Carminati, A. (2018). Root hairs increase rhizosphere extension and carbon input to soil. *Ann. Bot.* 121**,** 61-69. doi: 10.1093/aob/mcx127.

James, E.K., Reis, V.M., Olivares, F.L., Baldani, J.I., and Döbereiner, J. (1994). Infection of sugar cane by the nitrogen-fixing bacterium *Acetobacter diazotrophicus*. *J. Exp. Bot.* 45**,** 757-766. doi: 10.1093/jxb/45.6.757.

Robertson-Albertyn, S., Alegria Terrazas, R., Balbirnie, K., Blank, M., Janiak, A., Szarejko, I., et al. (2017). Root hair mutations displace the barley rhizosphere microbiota. *Front. Plant Sci.* 8**:**1094. doi: 10.3389/fpls.2017.01094

Schmidt, H., Nunan, N., Höck, A., Eickhorst, T., Kaiser, C., Woebken, D., et al. (2018). Recognizing patterns: Spatial analysis of observed microbial colonization on root surfaces. *Front. environ. sci* 6**:**61. doi: 10.3389/fenvs.2018.00061.

Schweiger, P.F., Robson, A.D., and Barrow, N.J. (1995). Root hair length determines beneficial effect of a *Glomus* species on shoot growth of some pasture species. *New Phytol.* 131**,** 247-254. doi: 10.1111/j.1469-8137.1995.tb05726.x.
